# A method for co-creation of an evidence-based patient workbook to address alcohol use when quitting smoking in primary care: a case study

**DOI:** 10.1186/s40900-018-0086-2

**Published:** 2018-02-05

**Authors:** Nadia Minian, Aliya Noormohamed, Laurie Zawertailo, Dolly Baliunas, Norman Giesbrecht, Bernard Le Foll, Jürgen Rehm, Andriy Samokhvalov, Peter L. Selby

**Affiliations:** 1 Centre for Addiction and Mental Health, 100 Stokes St, Toronto, ON M6J1H4 Canada; 2 Institute for Clinical Evaluative Sciences, Toronto, Ontario Canada; 3 Department of Family and Community Medicine, Psychiatry, University of Toronto, Toronto, Canada

**Keywords:** Evidence-based medicine, Community-sensitive, Participatory research, Co-creation, Patient engagement, Tobacco, Alcohol, Educational resources

## Abstract

**Plain English summary:**

The purpose of this paper is to describe a patient engagement event designed to create an educational workbook with smokers who drink alcohol at harmful levels. The goal was to create a workbook that combined scientific evidence with patients’ values, preferences, and needs. Fourteen adult smokers who drink alcohol were invited to the Centre for Addiction and Mental Health (CAMH) to take part in a four-hour event to help design the workbook with the CAMH research team. Participants provided their opinions and ideas to create an outline for the workbook, including activities, images, and titles. The workbook – called *Self-Awareness* – is currently being offered in a smoking cessation program in 221 primary care clinics across Ontario to help smokers quit or reduce their harmful alcohol use. The patient engagement event was a useful way to co-create educational materials that incorporate both scientific research and patient needs.

**Abstract:**

**Background**

Evidence-based medicine is the integration of best research evidence with clinical expertise and patient values. There are few methodologies on how to design evidence-based programs and resources to include patient values. The latter is an important aspect of patient-centered care, and is essential for patients to trust the recommendations and empower them as consumers to make informed choices. This manuscript describes a participatory research approach to design patient-facing educational materials that incorporate both evidence-based and community-sensitive principles. These materials are intended to support smokers to reduce or stop harmful alcohol consumption.

**Methods**

Adult smokers who report consuming alcohol were invited to a co-creation meeting at the Centre for Addiction and Mental Health’s Nicotine Dependence Service to guide the adaptation of evidence-based materials. The four-hour event consisted of individual reflections, group discussions, and consensus-building interactions. Detailed notes were taken and then incorporated into the material.

**Results**

Fourteen individuals participated in the event. The end product was a descriptive outline of an educational resource – entitled *Self-Awareness* – incorporating material from evidence-based workbooks and patient-driven features. Participants collaboratively selected the resource’s content, structure, and titles.

**Conclusions**

This model describes a participatory research method that emphasizes the value of the patient perspective; preliminary evidence finds this adaptation approach can increase the adoption of resources. The process described in this article could be replicated in other settings to co-create evidence-based resources, interventions, and programs that reflect the needs of the community.

**Trial registration**

ClinicalTrials.gov NCT03108144. Retrospectively registered 11 April 2017.

**Electronic supplementary material:**

The online version of this article (10.1186/s40900-018-0086-2) contains supplementary material, which is available to authorized users.

## Background

Two important and interrelated paradigms have been gaining traction in Canadian medicine; a renewed interest in promoting community-sensitive and patient-centered care [1], and creating programs and resources that are evidence-based [2]. Patient-centered, community-sensitive care seeks to create programs that are representative of the patients’ culture and values, thus enabling patients to feel comfortable, trusting, and respected [1, 3]. It also aims to empower patients as consumers, educating them about treatment options and allowing them to make informed choices if desired [4]. The goal of evidence-based practice is to incorporate (a) clinical expertise/expert opinion, (b) external scientific evidence, and (c) client/patient/caregiver perspective [5]; however there are few methodologies that detail how to incorporate community/patient values with scientific evidence. Researchers have shown that the terms “evidence-based medicine” and “patient-centered medicine,” are seldom used by the same authors, [6] and that there is a need for “evidence-based medicine to be more patient-centered …. (and) patient-centered medicine to be more evidence-based.” To-date, most of evidence-based medicine has focused on methods for conducting thorough systematic reviews and for reducing bias in clinical trials, [7] with little attention paid to the third concept – patient/community-centered strategies necessary for true evidence-based medicine [5, 6, 8, 9].

Canadian research funding agencies [10, 11],as well as several provincial government agencies [12–14] are trying to close this gap by promoting strategies that are evidence-informed and that engage patients in the delivery of health care. Despite such supportive policies, there are few existing resources that incorporate both of these concepts [6, 15]. In the winter of 2015 the Nicotine Dependence Service at the Centre for Addiction and Mental Health (CAMH) set out to develop an educational resource as part of a Canadian Cancer Society Research Institute-funded study entitled, “Personalized patient alerts and care pathways to prompt prevention interventions for **comb**ined **a**lcohol and **t**obacco users in primary care”, or, COMBAT [16]. The COMBAT trial is operationalized via the Smoking Treatment for Ontario Patients (STOP) program, an established smoking cessation program implemented at the primary care level in Ontario, Canada. The STOP program consists of up to 26 weeks of nicotine replacement therapy and behavioral counseling at no cost to the patient.

This educational resource was developed to be distributed by health care practitioners to smokers in the STOP program who report drinking alcohol above the Canadian Cancer Society cancer prevention alcohol consumption guidelines [17]. The purpose of the educational resource is to educate smokers about the multiplicative risk of aerodigestive cancers resulting from dual tobacco and alcohol consumption [18, 19]. This paper describes how the resource was developed in order to bridge the divide between evidence-informed and community-sensitive principles. Our aim was to make them as relevant and useful as possible for the community it is designed to serve.

## Methods

### Recruitment

A total of 215 individuals met our research team’s eligibility criteria for a half-day event at CAMH. Eligibility criteria stipulated that participants must be:

- Participant of the STOP program,
- Daily smoker trying to quit smoking
- Current alcohol user, as defined by reporting having consumed an alcoholic beverage in the past twelve months

Alcohol consumption in the past twelve months - versus a more comprehensive alcohol use measure as the AUDIT-C – was used to define “current alcohol user” in an effort to broaden the sample of participants amongst whom recruitment could take place. By involving individuals who smoke and consume alcohol in the design of the educational resource, our intention was to reduce threats to social validity [20] by ensuring the resource reflected our patients’ needs and interests. We successfully contacted 89 individuals by phone and/or email until reaching our goal of 20 individuals agreeing to participate. Our aim of reaching twenty individuals was based on research findings suggesting that a group size of no more than thirty captures relevant expertise while allowing for contributions from all participants [21], as well as logistic constraints (event room size, and budget). Of the individuals contacted who did not participate in the event, the most commonly cited reasons for not attending were that the event conflicted with their work schedule, or they were not interested in participating.

### Participants

Fourteen participants (67% of individuals that agreed to participate) attended the event. Of those participants that provided responses to a STOP program baseline questionnaire, exactly half were female and half male; 86% (12 individuals) reported having up to a college degree; 72%(10 individuals) reported an annual household income of $60,000 or less; 86% (12 individuals) reported smoking ten cigarettes or more per day; and 79% (11 individuals) reported consuming alcohol at hazardous levels, as defined by the AUDIT-C screening tool [22]. The AUDIT-C screening tool is an evidence-based, brief screen used to identify hazardous drinking, applying measures of quantity and frequency of alcohol consumption, as well as engagement in binge drinking [22]. There were no observable differences in demographic characteristics of individuals choosing to participate compared to those choosing not to participate in the event.

### Procedure

The patient engagement event was facilitated by two team members (NM and AN) who were responsible for guiding the discussion and eliciting relevant input from participants. Additionally, a support staff member (RR) was present to collect informed consent forms and field notes of the discussion, as well as to distribute refreshments and honoraria ($46 cash) to participants following the event. One facilitator (NM) was trained by ICA Canada in Group Facilitation Methods, and has over seven years of experience supporting communities to reflect o and develop their own appreciation of best practices, while at the same time being supported to share their knowledge of their health experiences and their local context. The other facilitator (AN) has specialized training in qualitative research focused on health behavior change and exploration.

The patient engagement event design was adapted from the Institute of Cultural Affairs Canada (ICA) consensus building methodology [23] to facilitate consensus among participants on the ideal resource for alcohol reduction among smokers. ICA methodology has the following five steps: (1) present the context and question, (2) brainstorm, (3) cluster, (4) name, (5) resolve. Our adaptation of ICA’s consensus building methodology included six steps:

### Step 1: Review and synthesize the evidence

We began by describing the purpose of the event, including describing the health risks associated with smoking and drinking alcohol above recommended cancer risk guidelines, and our end goal of co-creating an educational resource to encourage alcohol reduction. Our intention was to develop a resource that was largely modeled from existing, evidence-based workbooks that were chosen based on the following criteria: developed by reputable public health agencies, designed for a North American audience, focused on risky alcohol use, and based on research evidence. Reference workbooks included the National Institute on Alcohol Abuse and Alcoholism’s “Rethinking your Drinking” [24]; BC Partners for Mental Health and Addictions’ “Problem Substance Use Workbook” [25]; Capital Health Nova Scotia’s “My Choice: A Workbook For Making Changes” [26]; and College of Family Physicians of Canada and Canadian Centre on Substance Abuse’s “Drinking Smart: Your Health and Alcohol Consumption” [27]. AN presented common themes and features from the model workbooks to participants, who were given handouts to match the presentation materials. After brief individual reflection, participants were asked to use their handout to indicate which features they liked and disliked, along with descriptive rationale.

### Step 2: Individual reflection and small group brainstorming

After independently evaluating all features, participants were asked to gather into small groups to collectively reflect on the presented features, and brainstorm which concepts were missing from the list of evidence-based features, and which features should be prioritized for inclusion in the end product. Participants were asked to be as descriptive as possible when identifying features to be included (e.g., ideal design, wording, colors, formatting, etc.) Small groups were asked to share at least three new ideas with the larger group.

### Step 3: Achieving group consensus

First individually, then as a small group, participants were asked to reflect on all features presented over the course of the event and discuss their favorite features to be included in the resource, as well as any adaptations they felt were needed to make the features more applicable to their lives. As a small group, participants collaboratively chose ten to fifteen features they proposed for inclusion in the resource. This process ensured the resource’s features captured the diversity of ideas in the group and reflected participants’ preferences and values. They were asked to record each proposed feature on an index card, again with as much description as possible.

### Step 4: Clustering ideas

In this step, NM began collecting index cards from each group, a few at a time, and affixed them to a wall in front of the room. As more cards were collected, participants were asked to begin clustering cards by common themes, concepts, or purposes. This activity helped achieve “sections” or “chapters” of the educational resource.

### Step 5: Naming, ordering the clusters

Participants were encouraged to come to consensus on a title for each cluster of index cards, with support from the facilitators. These title cards would be used to name the resource’s sections. Participants were next asked to rearrange the clusters in the order they wished to see them presented in the resource; this process required group discussion to rationalize why certain sections would be better suited earlier versus later in the resource.

### Step 6: Naming the resource

Facilitator NM encouraged the group to reflect on the overall purpose of the educational resource, its newly-arranged content, and its primary audience. With this information, they came to consensus on a title for the educational resource.

Upon completion of the engagement event, participants completed a brief evaluation form to offer feedback on the event’s activities, moderators, and setting. Participants rated their agreement, on a 5-point Likert scale, with statements including, “This event offered a supportive and friendly environment” and “The instructor explained the activities clearly,” and responded to open-ended questions regarding overall impressions of the event.

## Results

Following individual, small group, and large group reflections, 29 index cards were presented to the moderators. These cards represented the evidence-based and/or participant-informed features that were identified for inclusion in the co-created educational resource on alcohol reduction among smokers. The cards were categorized by participants into five sections: 1. Internal and External Supports; 2. Self-Awareness Strategies; 3. Strategies for Success; 4. Dealing with Setbacks and Slips; and 5. Health Effects. Each section – described in greater detail below – was carefully selected to cover the range of topics discussed during the patient engagement event, including evidence-based principles for reducing risky alcohol use, and participant-driven features.

### Internal and external supports

During the engagement event, participants expressed the importance of having a support system easily accessible in the event of a crisis. Therefore they chose the first section of the resource to be a source of contact information for patients’ support networks, both external (e.g., provincial helplines) and internal, where patients can write in details of individuals in their personal support team.

### Self-awareness strategies

A consistent theme participants recognized across existing evidence-based resources was an opportunity to understand one’s own behaviors. For example, participants chose to include a daily tracking log for keeping record of cigarettes smoked and alcohol beverages consumed, an evidence-based strategy for encouraging behavior change [28]. Participants felt it was important for patients to understand their smoking and drinking patterns before planning steps to make a change.

### Strategies for success

During the event, participants expressed interest in evidence-based resources that offered guidance on how to make a change. Participants felt this section was a natural progression from the *Self-Awareness Strategies* section, since patients first understand their behaviors, then make a plan to change them. However, there were mixed feelings about whether that guidance should be more prescriptive or self-guided. As a result, the section – *Strategies for Success* – offers a combination of evidence-based tips for reducing alcohol consumption (e.g., drinking slowly, eating before drinking, etc.) and open-ended questions for patients to write in their preferred reduction strategies (e.g., “What are some reasons you can provide for not having a drink when offered one?”)

A participant-driven feature that was not initially presented, but rather arose from participant discussion, was a savings calculator – a tool to estimate the amount of money saved by reducing or quitting alcohol consumption. The tool was meant to act as a strategy for success by motivating patients to reduce alcohol use in order to save money for other activities of interest.

### Dealing with setbacks and slips

Participants appreciated that most evidence-based resources normalized setbacks and offered recommendations for addressing them. Similar to the *Strategies for Success* section of the resource, participants chose for this section to offer guidance to patients, and allow patients to decide what steps they will take to deal with relapse (e.g., “What will I tell myself if I have a slip?”)

### Health effects

**Fig. 1:**
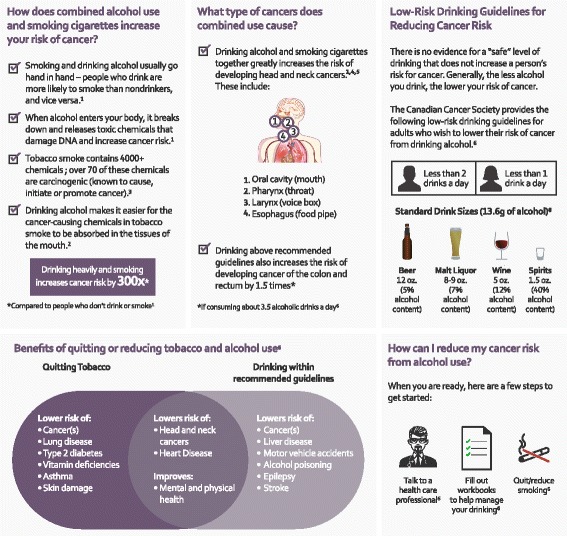
Educational workbook graphic example

The final section of the workbook was selected for inclusion by participants since most evidence-based resources to which they had exposure included health risks associated with the behavior being addressed. Participants felt patients would be less interested in this section compared to the behavioral exercises outlined in previous sections, and therefore chose for this section to be placed at the end of the resource. At participants’ request, our team depicted all health information in a visually-appealing, graphic format [see Fig. 1: Educational workbook graphic example].

All features were consolidated by a CAMH graphic design team into a 30-page alcohol reduction resource, entitled *Self-Awareness*. The resource was designed to meet standards of the Accessibility for Ontarians with Disabilities Act [29]. Refer to Additional files to view the finalized, co-created *Self-Awareness* workbook [see Additional file 1].

## Evaluation results

General impressions of the patient engagement event were overall positive. All participants agreed the organization and format of the session were good, and that the event offered a supportive and friendly environment. Nearly all participants (93%) agreed the small table discussions allowed their voice to be heard, and they were able to imagine how the session might apply in their community. In response to open-ended questions, one participant shared liking “how others can be in a large majority agreement and that basically everyone has the same concerns,” while others enjoyed “meeting new people” and the “knowledgeable and very inclusive” event. Though most participants expressed they wouldn’t have done anything differently to make the event better for them, one participant expressed the session “could have focused more on the connection between drinking and smoking and the related harms.”

## Resource in practice

In April 2016, 221 Ontario primary care clinics participating in the STOP smoking cessation program started offering this resource to eligible patients. In thirteen months, 4110 smokers in the STOP program reported consuming alcohol at levels exceeding the Canadian Cancer Society’s cancer prevention alcohol consumption guidelines), of which 1871 (46%) were offered the *Self-Awareness* educational resource by their health care practitioner. Utility and effectiveness of the resource in this real world setting is being evaluated.

## Discussion

The methodology described in this paper aims to diminish the growing gap between two prevailing paradigms in medicine – evidence-based practice and community-sensitive care – by creating a dynamic process that integrates the best research evidence with community values to create recommendations that will be widely adopted by the population and will achieve the best outcomes. The final resource offers unique features not currently available in existing, evidence-based resources. For example, the final resource reflects the interplay between tobacco and alcohol use, versus either health behavior alone. Compared to other evidence-based resources, [24, 25] it includes more visual graphics (i.e. infographics to communicate health effects of dual alcohol and tobacco use), as preferred by its intended users. This resource was also designed to meet the needs of its local audience by guiding patients to Ontario-based support services and defining alcohol quantities in metric units. As mentioned earlier, it also includes a unique, participant-designed tool to calculate savings of quitting or reducing alcohol use.

The patient engagement event approach described in this paper provides a rich description of the way a community interprets evidence-based recommendations, helps to identify key community concerns and assets, and describes how they can fit with community values, and how they have to be adapted in order to be adopted [4]. This collaborative research approach empowers patients to be involved in the care process, which is an especially important benefit for historically disenfranchised populations [4]. This approach fits well with the new health care environment that encourages the successful implementation of programs and resources that are both evidence-based and community-sensitive [12–14]. These methods provide information that may be used for both designing and improving resources and programs that will promote the uptake of research findings into routine healthcare [30]. Preliminary results of the COMBAT trial suggest this methodology created a resource that is more likely to be used by health care practitioners; 46% of health care practitioners are offering this patient-driven resource, compared to results from one study where approximately 3% of health care practitioners offered a resource to their eligible patients [31]*.*

Despite the benefits of this inclusive research methodology, there are some limitations to the process used. First, the patient engagement event was a one-time, half-day workshop, which reflects a limited snapshot of the participant group’s values and preferences. Allowing participants more time to reflect on the presented materials, or inviting additional participants to provide input on the resource, may have minimized the effect of this limitation. Second, one-third of individuals confirmed to participate in the event did not attend, presenting threat of selection bias among those that did attend. Lastly, although this resource was developed via participatory research [32], we cannot conclude it is of value to the patients for whom it was designed; similarly, we cannot conclude health care practitioners identify the resource as being of value for their patients. The resource’s effectiveness on patient outcomes is being evaluated as part of the COMBAT study.

## Conclusion

After the patient engagement event, CAMH was able to produce a 30-page evidence-based, community-sensitive workbook that includes information on the health effects of smoking and drinking, information on where participants can access help if needed, as well as several activities for participants to understand their health behaviours and develop a plan to change them. The systematic process used to develop this resource allowed for patients’ opinions and needs to be reflected, while reaching consensus in a larger group. This participatory research method showcases a more inclusive way of developing resources, where value is placed on both patients’ viewpoints and research evidence. This engagement event process could be replicated in other settings to co-create evidence-based resources, interventions, and programs that reflect the needs of the community.

## Additional file

Additional file 1: Co-created Self-Awareness workbook. (PDF 7746 kb)

## Abbreviations

CAMH: Centre for Addiction and Mental Health
COMBAT: Personalized patient alerts and care pathways to prompt prevention interventions for combined alcohol and tobacco users in primary care
ICA: Institute of Cultural Affairs Canada
STOP: Smoking Treatment for Ontario Patients
