# Supplementary material for: A method for co-creation of an evidence-based patient workbook to address alcohol use when quitting smoking in primary care: a case study
Source: Res Involv Engagem. 2018 Feb 5;4:4. doi: 10.1186/s40900-018-0086-2 (PMC5798180; doi:10.1186/s40900-018-0086-2)
Supplement: Additional file 1: — Co-created Self-Awareness workbook. (PDF 7746 kb) [file 40900_2018_86_MOESM1_ESM.pdf]

# SELF AWARENESS

camh NDS
Nicotine Dependence Service

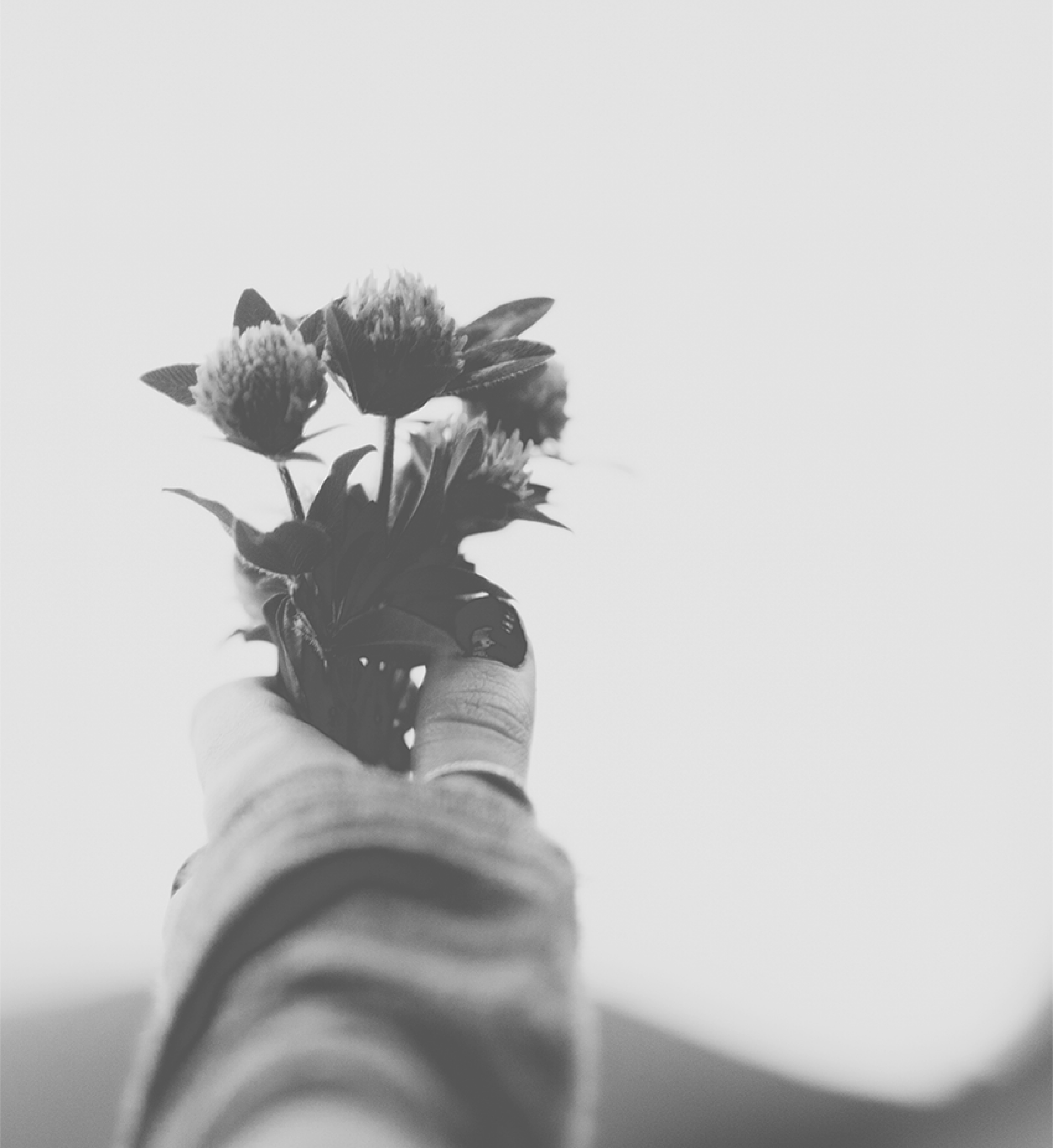

# TABLE OF CONTENTS


Introduction....3

- Is this workbook right for you? (AUDIT questionnaire)....4
- What does your score mean?....5

Internal and External Supports....6

- Immediate crisis support....6
- Your personal support system....7

Self-Awareness....8

- Your current drinking patterns....8
- How do your numbers compare to the low-risk drinking guidelines?....9
- Setting goals....10
- Life goals and alcohol use....11
- Cutting down or stopping drinking?....12
- Your change plan contract....13
- Keeping track of your drinking....14
- Daily tracking log....15
- Triggers....19

Strategies for Success....20

- Handling tough situations....21
- Saying "no"....21
- Reward yourself....22
- New activities....23
- Financial benefits of reducing or quitting drinking....24

Dealing with Setbacks and Slips....25

- Back on track plan....26

Health Effects....27

- Effects of high-risk drinking....27
- Alcohol use among smokers and cancer risk....28

Conclusion....29

References....30

# INTRODUCTION

Welcome to the Self-Awareness Workbook! This workbook was developed by the Centre for Addiction and Mental Health's Nicotine Dependence Service. In this workbook, we offer guidance, tips, tools, and resources to help reduce or quit drinking.

## Why is this workbook important?

Alcohol is often enjoyed when socializing with friends or family. Different people drink alcohol at different levels. Everyone's body responds differently to the alcohol they consume. Regardless of how much you drink, it is important to know the health effects linked to drinking alcohol, including cancers, liver damage, and high blood pressure. The harms of drinking are especially high among people who smoke. Compared to people who do not drink or smoke, those who drink and smoke heavily have a 300 times increased risk of cancer. Drinking alcohol makes it easier for cancer-causing chemicals in tobacco smoke to be absorbed in the tissues of the mouth and can greatly increase the risk of developing cancers of the head, neck and digestive system. This workbook is designed to help you understand your risks and address them in the best way for you.

## This workbook is not for everyone!

As a first step, we recommend completing the questionnaire on page 4 and referring to page 5 to figure out if this workbook is right for you.

If you have been found to drink at levels that indicate a high risk of alcohol dependence, this workbook is not appropriate for you. We recommend you stop drinking alcohol and seek guidance from a health professional (see page 6).

If you have been found to drink at risky levels but not alcohol dependent, this workbook is for you! This workbook is designed to help you create a healthy relationship with alcohol and reduce your risk of cancer associated with drinking alcohol and smoking. You can directly decrease your risk by reducing or quitting drinking. By following the steps listed throughout this workbook, we can help you set goals and make positive changes in your health and lifestyle.

There is no right or wrong way to use this workbook. Feel free to take your time with the activities and repeat them as often as you would like. We hope this workbook can help you become self-aware of your drinking behaviour and improve your quality of life.

Thank you for bringing us along on this journey. You can make the change, and we are here to help!

# AUDIT questionnaire

This questionnaire is to help you figure out if this workbook is appropriate for you or not. For each question in the chart below, CIRCLE the box that best describes your answer. Write the score for each circled box at the end of each line, then total these numbers at the bottom of the page.

NOTE: In Canada, a single drink serving contains 13.6 grams of ethanol or "pure" alcohol. Although the drinks below are different sizes, each one contains the same amount of pure alcohol and counts as a single drink:

| | | | | | | | |
|---|---|---|---|---|---|---|---|
| 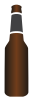 | 341 mL (12 oz.) of beer (about 5% alcohol) | 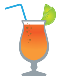 | 341 mL (12 oz.) of cider/cooler (about 5% alcohol) | 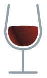 | 142 mL (5 oz.) of wine (about 12% alcohol) | 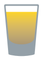 | 43 mL (1.5 oz.) of hard liquor (about 40% alcohol) |

| | Questions | 0 points | 1 point | 2 points | 3 points | 4 points | # pts. |
|---|---|---|---|---|---|---|---|
| 1 | How often do you have a drink containing alcohol? | Never | Monthly or less | 2 to 4 times a month | 2 to 3 times a week | 4 or more times a week | |
| 2 | How many drinks containing alcohol do you have on a typical day when you are drinking? | 1 or 2 | 3 or 4 | 5 to 6 | 7 to 9 | 10 or more | |
| 3 | How often do you have 5 or more drinks on one occasion? | Never | Less than monthly | Monthly | Weekly | Daily or almost daily | |
| 4 | How often during the last year have you found that you were not able to stop drinking once you had started? | Never | Less than monthly | Monthly | Weekly | Daily or almost daily | |
| 5 | How often during the last year have you failed to do what was normally expected of you because of drinking? | Never | Less than monthly | Monthly | Weekly | Daily or almost daily | |
| 6 | How often during the last year have you needed a first drink in the morning to get yourself going after a heavy drinking session? | Never | Less than monthly | Monthly | Weekly | Daily or almost daily | |
| 7 | How often during the last year have you had a feeling of guilt or remorse after drinking? | Never | Less than monthly | Monthly | Weekly | Daily or almost daily | |
| 8 | How often during the last year have you been unable to remember what happened the night before because of your drinking? | Never | Less than monthly | Monthly | Weekly | Daily or almost daily | |
| 9 | Have you or someone else been injured because of your drinking? | No | | Yes, but not in the last year | | Yes, during the last year | |
| 10 | Has a relative, friend, doctor, or other health care worker been concerned about your drinking or suggested you cut down? | No | | Yes, but not in the last year | | Yes, during the last year | |
| | | | | | | Total points = | |

*Note:* This questionnaire (the AUDIT) is adapted from the National Institute on Alcohol Abuse and Alcoholism, which reprinted the AUDIT with permission from the World Health Organization. To reflect drink serving sizes in Canada (13.6g of pure alcohol), the number of drinks in question 3 was changed from 6 to 5. A free AUDIT manual with guidelines for use in primary care settings is available online at www.who.org.

## What does your score mean?

Now that you have competed the AUDIT questionnaire (page 4), total up your scores for each answer. The maximum possible total is 40 points.

Compare your score with the chart below to help determine if this workbook is appropriate for you.

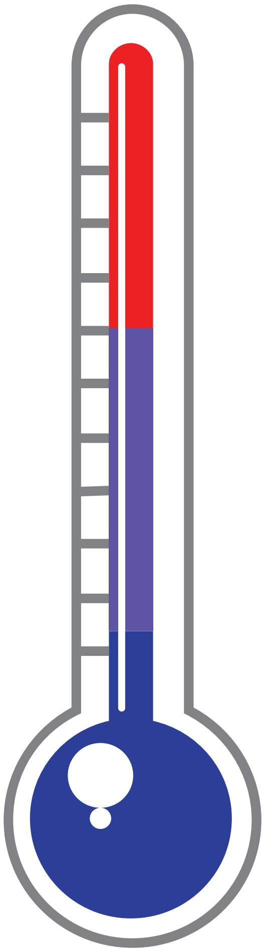

**Women and Men: 20+ points**

You are drinking at levels that indicate a high risk of alcohol dependence. This workbook may not be appropriate for you. We recommend you stop drinking alcohol and seek guidance from a health professional (see page 5 for support options).

**Women: 4-19 points**
**Men: 5-19 points**

You are drinking at levels that indicate risky drinking. We recommend you use this workbook to help you cut down or quit drinking alcohol.

**Women: 0-3 points**
**Men: 0-4 points**

You are drinking at low-risk levels. We encourage you to continue your progress to protect your health. You may choose to use this workbook to monitor your progress and increase your self-awareness.

### Why is there a difference between women's and men's scores?

Research shows that women start to have alcohol-related problems at lower drinking levels than men do. One reason is that, on average, women tend to weigh less than men. In addition, alcohol disperses in body water, and pound for pound, women have less water in their bodies than men do. So after a man and woman of the same weight drink the same amount of alcohol, the woman's blood alcohol concentration will tend to be higher, putting her at greater risk for harm.

Source: NIAAA. Rethinking Drinking: Alcohol & your health.

# INTERNAL AND EXTERNAL SUPPORTS

## Immediate crisis support

The following are a few resources to help you find support near you:

**ConnexOntario** provides free and confidential health services information for people experiencing problems with alcohol and drugs, mental illness or gambling.

**www.connexontario.ca**

**Drug and Alcohol Helpline** provides information about drug and alcohol addiction services in Ontario. The service is live answer 24/7, confidential and free.

**Call 1-800-565-8603**

**Or visit their website: www.drugandalcoholhelpline.ca**

**Smokers' Helpline** is a free, confidential service offering support and information about quitting smoking and tobacco use. There are 4 ways to get help from Smokers' Helpline:

1. Call 1 877 513-5333
2. Join the online quit program for tips, tools and support 24/7
3. Sign up for customized text messages offering support
4. Access free booklets on smoking and quitting

### Feeling depressed or anxious?

It's common for people to feel depressed or anxious when working through risky health behaviours. Mild symptoms may go away if you cut down or stop drinking. See your health care provider or a mental health professional if your symptoms don't go away or get worse. If you're having suicidal thoughts, call your health care provider or go to the nearest emergency room right away. Effective treatment is available to help you through this difficult time.

Source: NIAAA. Rethinking Drinking: Alcohol & your health.

## Your personal support system

You can also seek help from the people in your life and support groups. Try to find trustworthy people in your life to share in your journey, or find an established support group.

Who are some people that can help you in your journey to make a positive change?

Name:________________________________________________

Phone: ______________________ Email:_________________________________

Name:________________________________________________

Phone: ______________________ Email:_________________________________

Name:________________________________________________

Phone:______________________ Email:_________________________________

Name:________________________________________________

Phone:______________________ Email:_________________________________

Have a plan of how you want your support team to help you. Some options of what to ask for are:

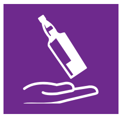

"Please don't offer me drinks."

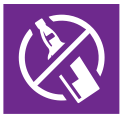

"Can we have an alcohol-free dinner party?"

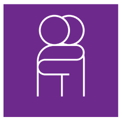

"Do you have any encouraging words to keep me going?"

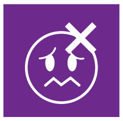

"If you see that I'm stressed, can you step in and offer help?"

What are some other ways you could ask your support network for help?

________________________________________________________________________

________________________________________________________________________

________________________________________________________________________

Consider joining mutual support groups. It may take some time to find a group that fits with your personality and interests. Try to be patient. By joining a group, you have a better chance of being successful in your journey to reduce or quit drinking.

# SELF-AWARENESS

This section focuses on some of the steps you can take to better understand your drinking patterns and to set goals for drinking at healthier levels. You have already completed the AUDIT questionnaire on page 4.

## Your current drinking patterns

This next exercise helps you understand your current drinking patterns by asking about your alcohol consumption over the past 7 days.

Reminder: In Canada, a single drink serving contains 13.6 grams of ethanol or "pure" alcohol. Although the drinks below are different sizes, each one contains the same amount of pure alcohol and counts as a single drink:

| | | | | | | | |
|---|---|---|---|---|---|---|---|
| | 341 mL (12 oz.)<br>of beer<br>(about 5% alcohol) | | 341 mL (12 oz.)<br>of cider/cooler<br>(about 5% alcohol) | | 142 mL (5 oz.)<br>of wine<br>(about 12% alcohol) | | 43 mL (1.5 oz.)<br>of hard liquor<br>(about 40% alcohol) |

1. Please circle yesterday's day of the week (e.g., if today is Monday, circle Sunday).
2. Starting on the day you circled (yesterday), please write down the number of drinks you had that day.
3. Continue following the arrows back. In each box, write the number of drinks you had on that day.
4. On days where you did not have anything to drink, please write "0." Do not leave any of the boxes blank.

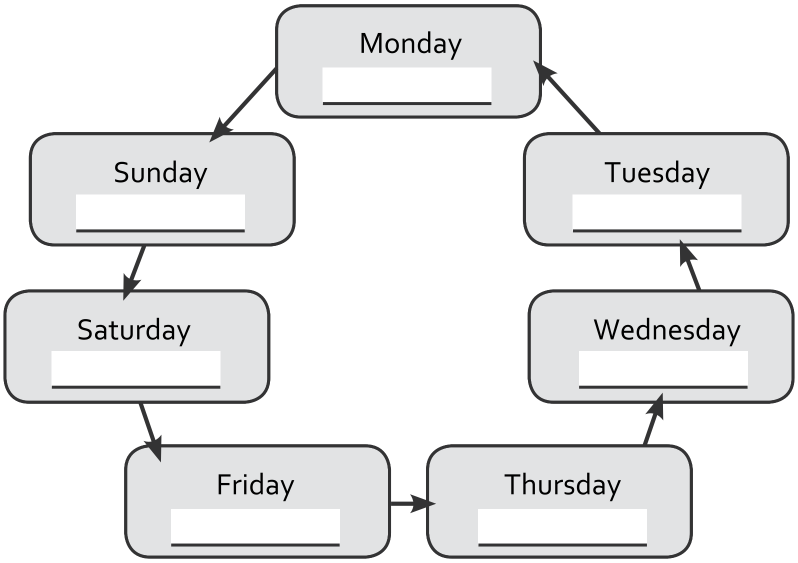


**Add up the # of drinks over the past 7 days:**

## How do your numbers compare to the low-risk drinking guidelines?

### What's "low-risk" drinking?

Generally, the less alcohol you drink, the more you reduce your health risks. In Canada, there are a few low-risk drinking guidelines established to help guide individuals to drink within safe limits. The Canadian Cancer Society (CCS) makes recommendations to reduce the risk of developing cancer as a result of drinking alcohol:

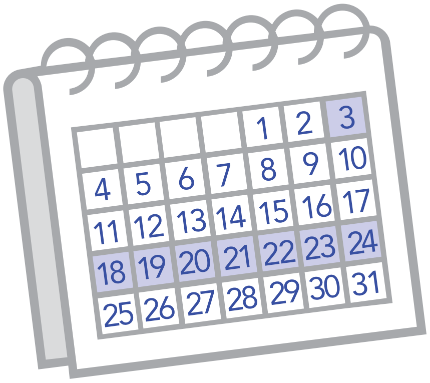


| Low-risk drinking limits | | Men | Women |
|---|---|---|---|
| | On any single DAY | Less than **2** drinks on any **day** | Less than **1** drink on any **day** |
| | Per WEEK | Less than **14** drinks in any **week** | Less than **7** drinks in any **week** |

You may see other low-risk drinking guidelines that allow for higher drinking levels on a given day or week. There is merit to different recommendations, but the Canadian Cancer Society guidelines are set to reduce your risk of developing several types of cancer, including mouth and throat cancers.

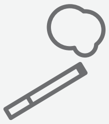

**The risk of developing mouth and throat cancers is higher for smokers than for non-smokers**

"Low risk" is not "no risk." Even within these limits, drinkers can have problems if they drink too quickly, have health problems, or are older. Based on your health and how alcohol affects you, you may need to drink less or not at all. If you are pregnant, you are strongly advised not to drink at all.

### What's "risky" drinking?

For healthy adults in general, drinking more than the single-day or weekly limits recommended by the Canadian Cancer Society is considered "risky" drinking.

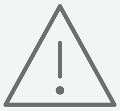

**About 1 in 4 smokers in Ontario drink at risky levels.**

It makes a difference both how much you drink on any day and how often you have a "heavy drinking day"—that is, more than 5 drinks in a day. The more drinks in a day and the more heavy drinking days over time, the greater the chances for health problems.

Source: NIAAA. Rethinking Drinking: Alcohol & Your Health; CAMH Internal Data.

## Setting goals

Before setting specific goals, it is important to first decide whether you want to cut down drinking or quit drinking. Both are legitimate goals. Your choice could depend on your personal circumstances.

| 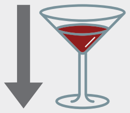<br>**It is recommended that you reduce drinking if you:** | 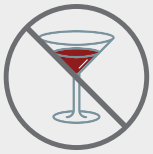<br>**It is recommended that you stop drinking if you:** |
| --- | --- |
| • Were not recommended to quit drinking by your health care provider<br>• Do not feel ready to quit drinking right now<br>• Drink at levels that don't negatively interfere with your:<br>» Personal relationships<br>» Work responsibilities<br>» Physical health<br>» Emotional health<br>» Social well-being<br>» Spiritual/ethical well-being | • Have been advised by your health care provider to quit drinking<br>• Have health problems such as liver disease or mental illness<br>• Are taking medications such as sedatives, painkillers or sleeping pills<br>• Have a personal or family history of drinking problems<br>• Have a family history of cancer or other risk factors for cancer<br>• Are pregnant, trying to get pregnant or breastfeeding (there is NO safe level of drinking in this situation)<br>• Will be operating vehicles such as cars, trucks, motorcycles, boats, snowmobiles, all-terrain vehicles or bicycles<br>• Are responsible for the safety of others at work or at home |

*Remember:* If you scored 20 points or higher on the AUDIT questionnaire (page 4), we recommend you stop drinking alcohol and seek guidance from a health professional (see page 6 for options).

Source: BC Partners for Mental Health and Addictions. Problem Substance Use Workbook; Capital Health Addiction Services. My Choice: A workbook for making changes.

# Life goals and alcohol use

Before thinking about your specific drinking goals, think more broadly about your life goals, at least for the next few months. What do you hope to accomplish? Will your current level of drinking help or hurt your goals?

| What would you want to accomplish over the next few months? | Would drinking at your current level make it EASIER or HARDER for you to reach these goals? | | |
|---|---|---|---|
| | Easier | No effect | Harder |
| 1. ______ | ☐ | ☐ | ☐ |
| 2. ______ | ☐ | ☐ | ☐ |
| 3. ______ | ☐ | ☐ | ☐ |
| 4. ______ | ☐ | ☐ | ☐ |
| 5. ______ | ☐ | ☐ | ☐ |

Source: College of Family Physicians of Canada and Canadian Centre on Substance Abuse. Drinking Smart: Your Health and Alcohol Consumption.

## Cutting down or stopping drinking?

It is ultimately your decision whether or not you want to change your drinking, and what your change goals would be. Weighing the pros and cons of cutting down and stopping drinking may help figure out which goal is right for you.

It is important to be honest with yourself during this activity, and that you take enough time to think of your answers.

**Pros:** What are some reasons why you might want to...

| Cut down drinking | Stop drinking |
| --- | --- |
| ______________________ | ______________________ |
| ______________________ | ______________________ |
| ______________________ | ______________________ |

*Examples could include: to lose weight, to sleep better, to be a better parent to my kids, to be happier*

**Cons:** What are some reasons why you might not want to...

| Cut down drinking | Stop drinking |
| --- | --- |
| ______________________ | ______________________ |
| ______________________ | ______________________ |
| ______________________ | ______________________ |

Take a look at your pros and cons. Highlight the points that you value the most.

Take a good look at your answers. What stands out as being very important to you? What is not as important as you thought it might be?

Now it's time to make a decision - which you can always change later! - about what will work best for you: cutting down or stopping drinking?

Write your decision here: **for my drinking goal, I have chosen to:**

## Your change plan contract

**My goal:**

☐ I want to drink no more than ___ drink(s) on any day and no more than ___ drink(s) per week (see page 9 for low-risk drinking guidelines)

*or*

☐ I want to stop drinking

**My start date:** I will start my change plan on this date: ______________________________

**My reasons:** My most important reasons to make these changes are:

______________________________

______________________________

**My strategies:** I will use these strategies to help make these changes (see page 20 for ideas):

______________________________

______________________________

**My support system:** The people who can help me are (names and how they can help) (see page 7 for ideas):

______________________________

______________________________

**Signs that I'm doing well:** I will know my plan is working if:

______________________________

______________________________

**My challenges and solutions:** Some things that might interfere and how I'll handle them (see pages 19-21 for ideas):

______________________________

______________________________

**Signature** ______________________________

*Optional,*

As your health care provider, I commit to providing professional support and guidance to help you make these changes:

**Health Care Provider Signature:** ______________________________

Source: NIAAA. Rethinking Drinking: Alcohol & Your Health.

## Keeping track of your drinking

Keeping track of your drinking can give you an idea of how much you drink and in which situations. Tracking your smoking alongside your drinking can help you get an overall picture of your behaviours and how they interact with each other.

You may find that keeping a drinking and smoking log helps you stick to your goals and monitor your progress. It may be inconvenient to track every drink, but committing to a tracking log may prove useful over time.

You will be asked to track how many "standard drinks" you have each day. As a reminder, a "standard drink" in Canada is measured as having 13.6 g of pure alcohol. The picture below may help give an idea of what that looks like in different types of drinks:

| Beer | Cider/Cooler | Wine | Hard Liquor |
|---|---|---|---|
| 341 mL<br>(12 oz.)<br>5% alcohol | 341 mL<br>(12 oz.)<br>5% alcohol | 142 mL<br>(5 oz.)<br>12% alcohol | 43 mL<br>(1.5 oz.)<br>40% alcohol |
| 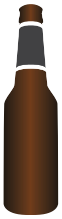 | 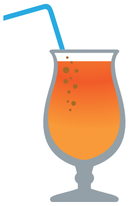 | 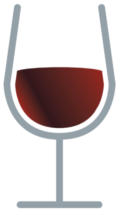 | 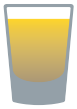 |

## Instructions:

1. At the top of your daily tracking log, write your drinking goal if you have decided on one (see page 13). Leave this space blank if you have not.

**For each day:**

2. Write the month and year at the top of the first column. In each space below, write the date for each day of the week.

3. Next, write the number of cigarettes you smoked that day in the "Total # of cigarettes for the day" column. If you didn't smoke on a day, then write "0" in the space.

4. In the next column, write the number of standard drinks you had that day in the "Total # of standard drinks for the day" column. If you didn't drink on a day, then write "0" in the space.

5. In the column "Urges to Drink?", rate the strength of the urge you felt to drink that day from 0–5 (0 being no urge, 5 being the strongest urge), and write this number in the space provided.

6. In the "Situation of the drinking or urge" column, write where you were and who you were with when you drank or had the urge to drink. You don't have to give the names of people or places, you can use initials or just put "friend" or "family".

7. In the last column, you can note what your thoughts and feelings were at the time you drank or had an urge to drink. There is space to make more notes related to any of your drinking situations or thoughts.

8. There is space at the bottom of the page, "Total # of drinks this week", to add up the drinks for the week.

   To make this tracking log as accurate as possible, it is important to record your drinking for the day at the end of each day, or if you forget, then as soon as you remember.

Source: Capital Health Nova Scotia. My Choice! Daily Drinking Tracking Log.

## Example: Daily tracking log

**GOAL: No more than _3_ drinks on a day and _15_ per week.**

| | Total # of cigarettes for the day | Total # of standard drinks for the day | Urges to drink? | Situation of the drinking or urge | Thoughts and feelings about your drinking or urges |
|---|---|---|---|---|---|
| **Feb / 2016 month/year** | **If you did not smoke on a day write "0"** | **If you did not drink on a day, write "0"** | **Rate the strength of the urge from 0-5** | **Write who you were with (or alone) where you were, and when** | **During the times you did drink or had urges to drink, write the feeling, thoughts you were having at the time. (e.g., you were bored, stressed, relaxed)** |
| Monday 18 | *6* | *0* | *3* | | *Fought really hard to resist a beer after dinner* |
| Tuesday 19 | *3* | *0* | *0* | | |
| Wednesday 20 | *12* | *3* | *5* | *Was watching TV* | *Was bored, nothing good on TV* |
| Thursday 21 | *9* | *0* | *4* | *Went to dad's house* | *Wanted to drink but didn't because dad is trying to quit* |
| Friday 22 | *15* | *5* | *5* | *Went out to bar with friends* | *Wanted to relax after work, and party* |
| Saturday 23 | *17* | *7* | *5* | *Had a get-together at my place* | *Was nervous that people were not having fun, and everyone else was drinking* |
| Sunday 24 | *8* | *0* | *2* | | |

Total # of drinks this week: *15*

**Additional notes:** *I had some pretty strong urges this week. I don't feel that I dealt with them in the best way since I drank over my daily goal a bunch on the weekend. But I did have some urges which I dealt with. I didn't drink on those days and I am happy about that. I noticed I drink more when I'm smoking…I'm going to ask the nurse about that.*

## GOAL: No more than ___ drinks on a day and ___ per week.

| | Total # of cigarettes for the day | Total # of standard drinks for the day | Urges to drink? | Situation of the drinking or urge | Thoughts and feelings about your drinking or urges |
|---|---|---|---|---|---|
| / month/year | If you did not smoke on a day write "0" | If you did not drink on a day, write "0" | Rate the strength of the urge from 0-5 | Write who you were with (or alone) where you were, and when | During the times you did drink or had urges to drink, write the feeling, thoughts you were having at the time. (e.g., you were bored, stressed, relaxed) |
| Monday | | | | | |
| Tuesday | | | | | |
| Wednesday | | | | | |
| Thursday | | | | | |
| Friday | | | | | |
| Saturday | | | | | |
| Sunday | | | | | |

Total # of drinks this week: ___________________________

**Additional notes:** ___________________________

___________________________

___________________________

## GOAL: No more than __ drinks on a day and ___ per week.

| | Total # of cigarettes for the day | Total # of standard drinks for the day | Urges to drink? | Situation of the drinking or urge | Thoughts and feelings about your drinking or urges |
|---|---|---|---|---|---|
| / month/year | If you did not smoke on a day write "0" | If you did not drink on a day, write "0" | Rate the strength of the urge from 0-5 | Write who you were with (or alone) where you were, and when | During the times you did drink or had urges to drink, write the feeling, thoughts you were having at the time. (e.g., you were bored, stressed, relaxed) |
| Monday | | | | | |
| Tuesday | | | | | |
| Wednesday | | | | | |
| Thursday | | | | | |
| Friday | | | | | |
| Saturday | | | | | |
| Sunday | | | | | |

Total # of drinks this week: ___________________________

**Additional notes:** ____________________________________________

____________________________________________

____________________________________________

## Triggers

Triggers are the patterns that usually occur before or around the time you drink. Triggers can be people, places, activities, etc. For example, you might learn that you drink more when you are smoking, or when you visit your neighbour on the weekend. In these cases, smoking and visiting your neighbour on the weekend are all triggers.

It is important to know what your triggers are and to have a plan for dealing with them in a way other than drinking. This will give you a better chance of achieving your goals.

## Identifying my Triggers

Note: *To complete this exercise, you'll need to have completed the Daily tracking log (page 17) for at least two weeks, preferably longer. The longer you have completed the Daily tracking log, the more easily you'll be able to see patterns in your drinking behaviour.*

Take a look at your entries in the Daily tracking log so far. Look at the days that you drank more than your daily goal and the days you had the strongest urge to drink. Place a mark beside these days or highlight them. Now look at what you have written in the last two columns on the days you highlighted (the ones titled "Situation of the drinking or urge" and "Thoughts and feelings about your drinking or urges"). Copy what you wrote on those days here:

______________________________________________________________________________

______________________________________________________________________________

______________________________________________________________________________

______________________________________________________________________________

______________________________________________________________________________

Source: Capital Health Addiction Services. My Choice: A workbook for making changes.

# STRATEGIES FOR SUCCESS

Follow these tips to help you reduce the amount you drink or to quit drinking alcohol. By following these steps and coming up with strategies of your own, you can improve your health and reduce your risk of developing certain cancers:

| Steps to reduce drinking | Steps to quit drinking |
|---|---|
| Plan ahead and set a limit on the amount you will drink (see page 13). | If you are thirsty, use a non-alcoholic drink like water to quench your thirst. |
| If you are thirsty, use a non-alcoholic drink like water to quench your thirst. | Do an activity when you are out with friends to help distract you, like playing pool or dancing. Drink water during these activities. |
| Alternate alcoholic beverages with non-alcoholic drinks like water, soft drinks or juice. | Don’t use alcohol to cope with stress. Go for a walk, take a bath, read a book or listen to some of your favourite music to help you relax. |
| Drink alcoholic beverages slowly. | Have something to eat or a non-alcoholic drink. |
| Space out your alcoholic drinks. | Leave the situation. |
| Try to drink lower alcohol content drinks, or mix alcoholic drinks with water, low-calorie soft drinks, club soda or juice. | Use relaxation techniques, such as breathing or meditating. |
| Don’t refill your glass until it is empty. | Take the money you would have used to drink and put it towards something you have always wanted (vacation, new outfit, night out, etc.) |
| Don’t “free pour” drinks because you may be drinking more than you think. Measure the amount of alcohol when you pour drinks so you know how much you are drinking. | Make a list of why you don’t want to drink and refer to it when triggers pop up. |
| Don’t try to keep up with your friends “drink for drink” because everyone handles alcohol differently. | |
| Avoid playing drinking games because they can make you drink large amounts of alcohol in a short time. | |
| Eat before and while you are drinking. | |
| Snack with your drinks, but avoid salty snacks like potato chips. Salty snacks can make you thirsty and may cause you to drink more and drink quickly. | |
| Set small, reasonable goals for yourself. If you are a regular drinker, try to set a goal of a few alcohol-free days each week. Small goals like this will be more successful than trying to cut out alcohol all at once. | |

Source: Canadian Cancer Society. Alcohol: Reducing your risk.

## Handling tough situations

From time to time, you will find yourself in situations where you want to drink or where other people are drinking. You may have learned about some of these situations when identifying your triggers on page 19.

You have many choices of how to respond to these situations, including the "Strategies for Success" examples provided on page 20.

Which strategies do you think would work best for you?

## Saying "no" ⃠

When you are faced with an urge to drink or other people are drinking around you, having a plan of how to say "no" can be extremely helpful. Some reasons for not drinking that you could use include:

| | | |
|---|---|---|
| *"I am the designated driver today"* | *"I promised my kids I wouldn't drink tonight"* | *"No thanks, I'm trying to cut down"* |

What are some reasons you can provide for not having a drink when offered one?

## Reward yourself

Committing to a healthier lifestyle deserves a reward! When you drink less, you spend less time on alcohol-related activities (e.g., sitting at a bar) and earn time to spend on other things you enjoy or have always wanted to do but never had the time.

For example, you can:

- Spend more time with your children
- Volunteer at a local shelter or hospital
- Start a new hobby
- Join a program at the community centre

Planning ahead is important. When you are bored, you may be tempted to fill your time with familiar activities. In this way, boredom can act as a trigger! When you plan ahead, you are giving yourself a chance to fill your time with a new or different activity.

What would you do if you had **MORE TIME?**

For inspiration, explore some options on the next page!

## New activities

### What are some activities you would like to try?

| | | | |
|---|---|---|---|
| Poetry | Keeping a diary | Traveling | Getting a pet |
| Running | Learning magic tricks | Clothesmaking | Floral arranging |
| Tree planting | Wood-carving | Needlepoint | Paint-by-numbers puzzle |
| Aromatherapy | Ceramics | Hiking | Working in the garden |
| Doing crosswords puzzles | Reading novels | Birdwatching | Meeting with friends |
| Writing short stories | Swimming | Getting a massage | Meditating |
| Cooking | Singing | Fishing | Photography |

| I will definitely try this! | I *might* try this... | No thank you! |
|---|---|---|
| | | |

Source: BC Partners for Mental Health and Addictions. Problem Substance Use Workbook.

## Financial benefits of reducing or quitting drinking

Did you know the average Ontarian (15 years and older) spends $635.10 each year on alcoholic beverages?

How do you compare? You can use this guide to help you calculate how much you spend on alcoholic beverages in a year:

### Alcohol

| | | | |
|---|---|---|---|
| A | On average, how many days per week do you drink alcohol?<br>*Sample response: 4 days* | ______________________ | |
| B | On a typical drinking day, how many drinks do you have?<br>*Sample response: 2 drinks* | ______________________ | × |
| C | What's the average price you spend per drink?<br>*Sample response: $4* | $______________________ | × |
| | Your spending average per week= A × B × C<br>*Sample calculation: 4 × 2 × $4 = $32* | $______________________ | |
| | Your spending average per week × 52 weeks =<br>Your spending average per year<br>Sample calculation: *$32 × 52 = $1664* | $______________________ | |

By quitting drinking, you could save $ ____________________ per year! Think about all the possibilities.

What would you do if you had **MORE MONEY?**

______________________________________________________________

______________________________________________________________

______________________________________________________________

______________________________________________________________

______________________________________________________________

To learn more, you can use the "Alcohol Spending Calculator" at the National Institutes of Health website: http://rethinkingdrinking.niaaa.nih.gov/tools/Calculators/alcohol-spending-calculator.aspx

You can also calculate how much you spend on cigarettes with The Lung Association's "Smoking cost calculator" at https://www.quitnow.ca/tools-and-resources/calculate-your-savings

Source: Statistics Canada. (2014, March 31). Sales of alcoholic beverages per capita, 15 years and older.

# DEALING WITH SETBACKS AND SLIPS

Sometimes it can be tough to stick to your goals. It is common to face challenges along a journey to make a positive change. It is also normal to take more time to make a change than you expected.

Changing your drinking behaviour is a process that does not have a set timeline; it will take longer for some than others. If you have a setback, you might be tempted to say, “Oh well, I failed to meet my goal so I guess I am back to square one.” **Stop right there.**

A setback does NOT put you back at the beginning. Seeing a setback or slip as only a turn in the path or a bump in the road, you are thinking positively and giving yourself the best chance of success.

## There is a difference between a slip (setback) and a relapse.

| | |
|---|---|
| A **slip** is considered any alcohol use above your drinking goal (i.e., one drink is a slip if your goal is to stop drinking. If your goal on a day is to drink less than two drinks and you had four, you’ve had a slip.) | A **relapse** is returning to the same level and pattern of use as before. For example, if you return to daily drinking, not sleeping well, arguing with your partner, and missing work when your goal was abstinence, you’ve relapsed. |

When you know the difference between a slip and a relapse, you are more likely to bounce back from a slip. You will know how to avoid turning that slip into a relapse and will feel more prepared to get back on track.

If you have a slip, don’t panic. Take some time to reflect on your journey so far by reading through this workbook and revisiting your goals and motivations for changing (pages 11 and 13).

*Remember:* A slip does not mean you lost all the progress you have made. By completing this workbook, you already have a better understanding of yourself and how to get back on track.

Source: Capital Health Nova Scotia. My Choice A Workbook For Making Changes.

## Back on track plan

Slips can pop up at any moment and very quickly turn into something dangerous and uncontrollable. Preparing a "Back on track plan" can help you feel ready to respond to a slip and prevent it from turning into a relapse.

**What I will tell myself if I have a slip?**

*Example: "Don't worry. I will do better next time."*

________________________________________

________________________________________

________________________________________

________________________________________

________________________________________

**What steps will I take if I have a slip?**

*Example: "Take a hard look at my triggers and see what went wrong, then try to avoid my triggers next time they pop up."*

________________________________________

________________________________________

________________________________________

________________________________________

________________________________________

## Effects of high-risk drinking

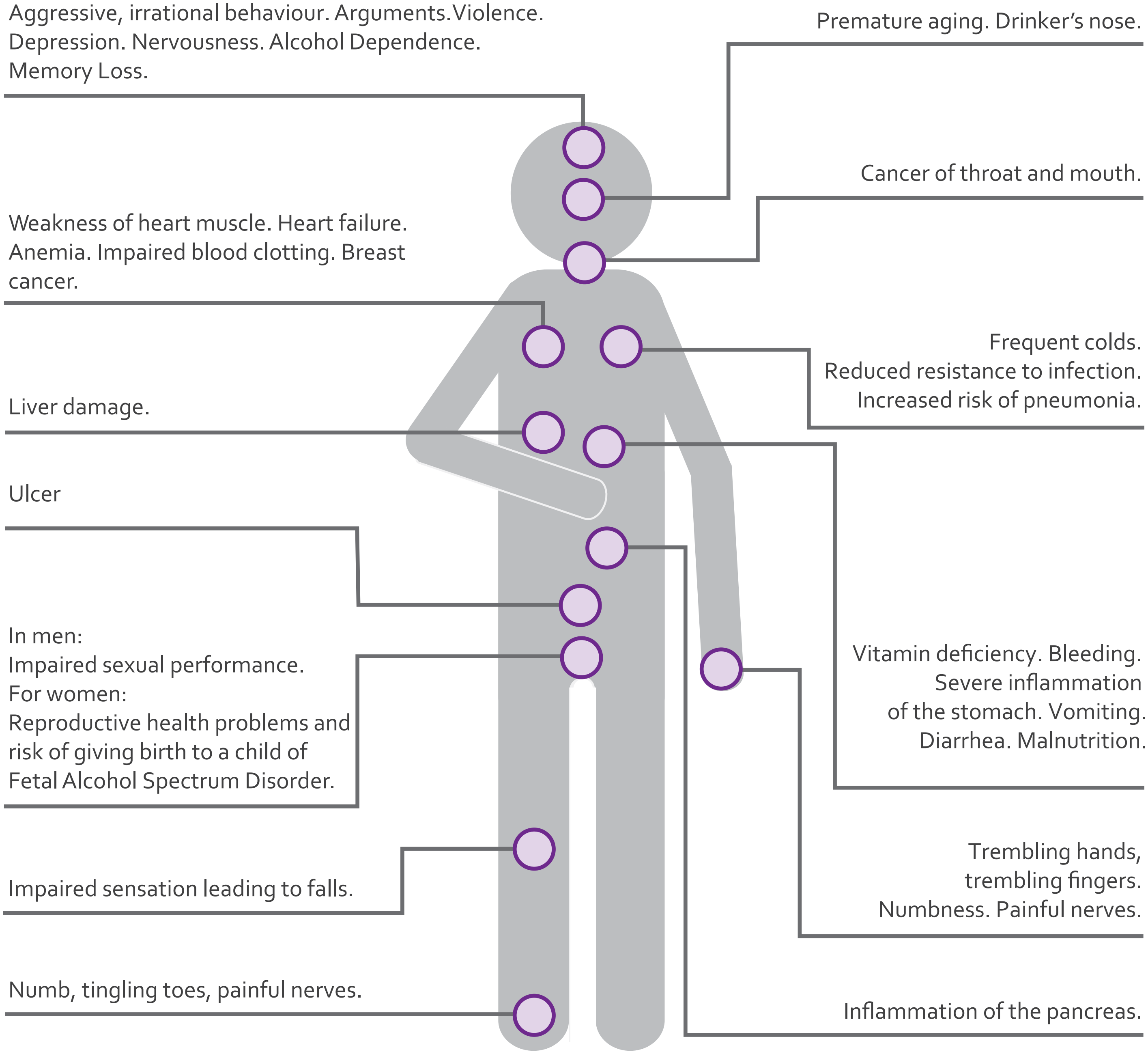


Adapted from Babor, T.F., et al. (2001). The alcohol use disorders identification test: Guidelines for use in primary care (p. 7). 2nd ed. Geneva: World Health Organization.

## Alcohol use among smokers and cancer risk

### How does combined alcohol use and smoking cigarettes increase your risk of cancer?

- ☑ Smoking and drinking alcohol usually go hand in hand – people who drink are more likely to smoke than nondrinkers, and vice versa.
- ☑ When alcohol enters your body, it breaks down and releases toxic chemicals that damage DNA and increase cancer risk.
- ☑ Tobacco smoke contains 4000+ chemicals ; over 70 of these chemicals are carcinogenic (known to cause, initiate or promote cancer).
- ☑ Drinking alcohol makes it easier for the cancer-causing chemicals in tobacco smoke to be absorbed in the tissues of the mouth.

**Drinking heavily and smoking increases cancer risk by 300x***

*Compared to people who don't drink or smoke

### What type of cancers does combined use cause?

- ☑ Drinking alcohol and smoking cigarettes together greatly increases the risk of developing head and neck cancers. These include:

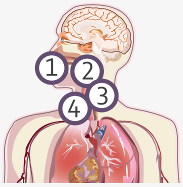


1. Oral cavity (mouth)
2. Pharynx (throat)
3. Larynx (voice box)
4. Esophagus (food pipe)

- ☑ Drinking above recommended guidelines also increases the risk of developing cancer of the colon and rectum by 1.5 times*

*If consuming about 3.5 alcoholic drinks a day

### Low-Risk Drinking Guidelines for Reducing Cancer Risk

There is no evidence for a "safe" level of drinking that does not increase a person's risk for cancer. Generally, the less alcohol you drink, the lower your risk of cancer.

The Canadian Cancer Society provides the following low-risk drinking guidelines for adults who wish to lower their risk of cancer from drinking alcohol.

| | |
|---|---|
| 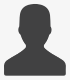 Less than 2 drinks a day | 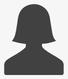 Less than 1 drink a day |

**Standard Drink Sizes (13.6g of alcohol)**

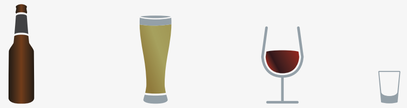

| Beer | Cider/Cooler | Wine | Spirits |
|---|---|---|---|
| 12 oz. (5% alcohol content) | 12 oz. (5% alcohol content) | 5 oz. (12% alcohol content) | 1.5 oz. (40% alcohol content) |

### Benefits of quitting or reducing tobacco and alcohol use

**Quitting Tobacco**

**Drinking within recommended guidelines**

**Lower risk of:**
- Cancer(s)
- Lung disease
- Type 2 diabetes
- Vitamin deficiencies
- Asthma
- Skin damage

**Lowers risk of:**
- Head and neck cancers
- Heart Disease

**Improves:**
- Mental and physical health

**Lowers risk of:**
- Cancer(s)
- Liver disease
- Motor vehicle accidents
- Alcohol poisoning
- Epilepsy
- Stroke

### How can I reduce my cancer risk from alcohol use?

When you are ready, here are a few steps to get started:

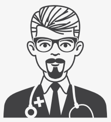
Talk to a health care professional

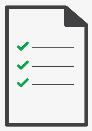
Fill out workbooks to help manage your drinking

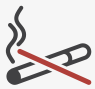
Quit/reduce smoking

# CONCLUSION

Congratulations! You have reached the end of your journey with us. Thank you for taking the time to read through this workbook and completing the suggested activities. Hopefully this workbook has been a useful tool in becoming more self-aware about your relationship with alcohol and given you the strategies and support you need to be successful in reducing your alcohol intake or quitting altogether.

This is a challenging journey to undertake so we understand if you may not have been successful the first time around. But deciding to read through and complete this workbook is a success in itself! We hope you re-visit this resource to review, revise, or reflect on your growth.

You may find it helpful to review your progress with your health care practitioner. If you feel you need more support along the way, browse through your support options again on page 7.

We wish you all the best in your journey towards a more positive, healthy lifestyle !

**This workbook is an adaptation of four evidence-based workbooks:**

- *BC Partners for Mental Health and Addictions. Problem Substance Use Workbook.*
- *Capital Health Nova Scotia. My Choice A Workbook For Making Changes.*
- *College of Family Physicians of Canada and Canadian Centre on Substance Abuse. Drinking Smart: Your Health and Alcohol Consumption.*
- *NIAAA. Rethinking Drinking: Alcohol & Your Health.*

Adaptations were shaped by a community-informed engagement event with clients enrolled in a smoking cessation program at the Centre for Addiction and Mental Health (CAMH) and reported drinking alcohol above the Canadian Cancer Society's low-risk drinking guidelines.

The development of this workbook was funded by the Canadian Cancer Society (Grant #703404)

# REFERENCES


Babor, T.F., et al. (2001). The alcohol use disorders identification test: Guidelines for use in primary care (p.7). 2nd ed. Geneva: World Health Organization.

Bagnardi, V., Blaniardo, M., LaVecchia, C., and Carrao G. (2001) A meta-analysis of alcohol drinking and cancer risk. British Journal of Cancer, 85:1700–1705.

BC Partners for Mental Health and Addictions. Problem Substance Use Workbook. Retrieved from http://www.heretohelp.bc.ca/publications/toolkits

Canadian Cancer Society. Alcohol. Retrieved from http://www.cancer.ca/en/prevention-and-screening/live-well/alcohol/?region=on

Canadian Cancer Society. Alcohol Rl;isk Factors. Retrieved from http://www.cancer.ca/en/cancer-information/cancer-101/what-is-a-risk-factor/alcohol/?region=on

Cancer Care Ontario. Cancer Risk Factors in Ontario: Alcohol Report. Retrieved from https://www.cancercare.on.ca/ocs/csurv/info/alcoholreport

Capital Health Nova Scotia. My Choice A Workbook For Making Changes. Retrieved from http://www.cdha.nshealth.ca/

Capital Health Nova Scotia. My Choice! Daily Drinking Tracking Log. Retrieved from: http://www.cdha.nshealth.ca/mental-health-and-addictions/patients-clients-and-visitors/patient-resources

College of Family Physicians of Canada and Canadian Centre on Substance Abuse. Drinking Smart: Your Health and Alcohol Consumption. Retrieved from http://www.sbir-diba.ca/resources/patient-resources

Health Canada. (2011, March 30).Tobacco Scientific Facts. Retrieved from www.hc-sc.gc.ca/hc-ps/tobac-tabac/fact-fait/facts-faits-eng.php

IARC. (2014). European Code Against Cancer - 12 Ways To Reduce Your Cancer Risk. Retrieved from http://cancer-code-europe.iarc.fr/index.php/en/

NIAAA. Alcohol Spending Calculator. Retrieved from http://rethinkingdrinking.niaaa.nih.gov/tools/Calculators/alcohol-spending-calculator.aspx

NIAAA. Assessing Alcohol Problems: A Guide for Clinicians and Researchers – Timeline Followback. Retrieved from: pubs.niaaa.gov

NIAAA. Helping Patients Who Drink Too Much. Retrieved from http://www.niaaa.nih.gov/guide

NIAAA. Rethinking Drinking: Alcohol & Your Health. Retrieved from www.RethinkingDrinking.niaaa.nih.gov

Pelucchi, C., Gallus, S., Garavello,W., Bosetti, C., & La Vecchia, C. (2006). Cancer risk associated with alcohol and tobacco use: Focus on upper aero-digestive tract and liver. Alcohol Research & Health, 29(3), 193-198.

Statistics Canada. (2014, March 31). Sales of alcoholic beverages per capita, 15 years and older. Retrieved from: http://www.statcan.gc.ca/daily-quotidien/150504/t150504a003-eng.htm#fn01

Szymańska, K., Hung, R. J.,Wünsch-Filho, V., Eluf-Neto, J.,Curado, M. P., Koifman, S., & Brennan, P. (2011). Alcohol and tobacco, and the risk of cancers of the upper aerodigestive tract in Latin America: a case–control study. Cancer Causes & Control, 22(7), 1037-1046.

The Lung Association. Smoking Cost Calculator. Retrieved from https://www.quitnow.ca/tools-and-resources/calculate-your-savings
